# Cardiovascular complications in chronic active Epstein–Barr virus disease: a case report and literature review

**DOI:** 10.3389/fped.2024.1480297

**Published:** 2025-01-10

**Authors:** Zhiyuan Feng, Hongyu Duan, Lin Wang, Huan Yu, Kaiyu Zhou, Yimin Hua, Chuan Wang, Xiaoliang Liu

**Affiliations:** ^1^ Key Laboratory of Birth Defects and Related Diseases of Women and Children of Ministry of Education (MOE), West China Institute of Women and Children’s Health, Key Laboratory of Development and Diseases of Women and Children of Sichuan Province, Department of Pediatrics, Department of Pediatric Cardiology, West China Second University Hospital, Sichuan University, Chengdu, Sichuan, China; ^2^ Department of Pediatrics, Longquanyi District of Chengdu Maternity & Child Health Care Hospital, Chengdu, Sichuan, China; ^3^ Department of Pediatric Cardiology, West China Second University Hospital (WCSUH)-Tianfu·Sichuan Provincial Children’s Hospital, Meishan, Sichuan, China

**Keywords:** chronic active Epstein-Barr virus infection, coronary artery aneurysm, valsalva sinus aneurysm, aortic lesions, EBV-associated hemophagocytic lymphohistiocytosis

## Abstract

**Background:**

Cardiovascular involvement is a rare but severe complication of Epstein–Barr virus (EBV) infections. Patients with chronic active EBV (CAEBV) are at increased risk of developing cardiovascular complications and have a poor prognosis. Here, we report the rare case of a pediatric patient with CAEBV and EBV- hemophagocytic lymphohistiocytosis (HLH) complicated with a giant coronary artery aneurysm (CAA) and thrombosis, a giant Valsalva sinus aneurysm, and ascending aorta dilation seven years after the disease onset.

**Case presentation:**

A previously healthy 3-year-old girl was initially misdiagnosed as presenting incomplete Kawasaki disease complicated by coronary artery lesions (CALs) for which she received intravenous immunoglobulin and aspirin therapy. Subsequently, she was transferred to our hospital, where we diagnosed her as having a primary EBV infection. After acyclovir therapy, her clinical symptoms resolved with negative EBV-DNA, and she was discharged home with aspirin treatment for the remaining CALs. However, she did not have regular follow-ups after that. Seven years later, the 10-year-old girl developed a prolonged fever and fatigue, and she was diagnosed as presenting CAEBV and EBV-associated hemophagocytic lymphohistiocytosis (EBV-HLH) due to the presence of a high EBV-DNA load, prolonged fever, splenomegaly, bicytopenia, hypertriglyceridemia, hypofibrinogenemia, hemophagocytosis, low NK-cell activity, and increased levels of ferritin and soluble CD25. The echocardiography images showed giant left and right coronary artery aneurysms, a giant Valsalva sinus aneurysm, and ascending aorta dilation. Her parents agreed to a therapy with intravenous immunoglobulin, methylprednisolone, antiplatelet, and anticoagulant, but not to the standard therapy of EBV-HLH. However, the cardiovascular complications, including CAAs and thrombosis, Valsalva sinus aneurysm, and aorta lesions, did not resolve. Three weeks later, the patient was finally discharged home asymptomatic. Unfortunately, one month after discharge, the fever recurred the girl. The guardian had refused treatment and took the patient home due to economic difficulties. During our subsequent follow-up visit, the girl subsequently passed away.

**Conclusions:**

We reported the case of a pediatric patient with EBV infection who developed rare and fatal cardiovascular complications (CAAs and thrombosis, Valsalva sinus aneurysm, and aortic lesions) seven years after the onset of the infection. Clinicians should be aware of these complications during the long-term follow-up of patients with EBV infection, especially in patients with CAEBV and/or EBV-HLH.

## Introduction

Epstein-Barr virus (EBV), a member of the *γ*-herpesviruses, may cause persistent, lifelong, latent infections (1). Children with primary EBV infection are usually asymptomatic, but they sometimes develop infectious mononucleosis (IM), which resolves spontaneously after the emergence of EBV-speciﬁc immunity. EBV infection in children is considered a self-limiting disease with a favorable prognosis (2). However, patients with an immune deficiency or impairment may develop an aggressive EBV infection that can result in chronic active EBV (CAEBV) disease, hemophagocytic lymphohistiocytosis (HLH), and tumors. CAEBV is characterized by an unusual pattern of anti-EBV antibodies and by chronic or recurrent symptoms mimicking an infectious mononucleosis that persist for a long time. Peripheral blood samples of patients with CAEBV have high virus loads. CAEBV is a high-mortality, high-morbidity disease with life-threatening complications. The pathogenesis of CAEBV involves the clonal expansion of EBV-infected T cells and NK cells (3). EBV can infect lymphocytes and other tissues leading to cardiovascular complications. Approximately 8.5% of patients with CAEBV present with coronary artery lesions (CALs) (4). In contrast, the incidence of CALs in Kawasaki disease is only 4% after intravenous immunoglobulin (IVIG) treatment (5). In addition to CALs, EBV infection may also cause myocarditis, valvular heart disease, aortic lesions, heart failure, and pulmonary artery hypertension (PAH) (6). However, we found only two reports of patients with CAEBV presenting with Valsalva sinus involvement at the onset of their disease (7, 8).

In here, we report the uncommon case of a pediatric patient with EBV infection, whose disease progressed to CAEBV and EBV-HLH with fatal cardiovascular involvement seven years after the infection onset. The cardiovascular findings included a giant coronary artery aneurysm and thrombosis, giant Valsalva sinus aneurysm, and a dilated ascending aorta.

## Case presentation

A previously healthy 3-year-old girl (Chinese, Han ethnicity) was admitted to the local hospital with a 10-day fever of unknown origin. The physical examination revealed the presence of rash, oral changes, and cervical lymphadenopathy. Laboratory test results showed elevated levels of C-reaction protein (CRP) at 14 mg/L (normal reference value: <8 mg/L), ALT at 226 U/L (normal range < 49 U/L), and AST at 230 U/L (normal range < 40 U/L), as well as an abnormal erythrocyte sedimentation rate (ESR) at 34 mm/h (normal reference value: 0–26 mm/h). The blood routine examination showed normal level of white blood cells (WBC, 5.1 × 10^9^/L, normal range: 4.3–11.3 × 10^9^/L), neutrophils (3.9 × 10^9^/L, normal range: 0.86–6.03 × 10^9^/L), lymphocytes (1.26 × 10^9^/L, normal range: 1.47–5.35 × 10^9^/L), platelets (PLT, 203 × 10^9^/L, normal range: 128–420 × 10^9^/L), hemoglobin (HB, 116 g/L, normal range: 108–144 g/L), and no atypical lymphocytes were detected. Echocardiography images revealed a left coronary artery aneurysm (LCA, 3.7 mm; *z* score, 4.60) and left anterior descending (LAD, 2.6 mm; *z* score, 3.07), left circumflex branch (LCX, 1.8 mm; *z* score, 1.29), right coronary artery (RCA, 3.4 mm; *z* score, 5.32) aneurysms. A primary diagnosis of incomplete Kawasaki disease (iKD) was proposed based on these findings. On the 11th day after the onset of the fever, she received IVIG (2 g/kg) as a single intravenous infusion and aspirin (30 mg/kg/day) treatment. However, the fever and cervical lymphadenopathy persisted beyond 48 h after the IVIG infusion. The patient was transferred to our hospital on the 14th day after the fever onset. The physical examination upon arrival revealed the presence of fever, tonsillitis with membrane formation, cervical lymphadenopathy, splenomegaly, and hepatomegaly. Symptoms such as coughing, headaches, vomiting, diarrhea, or shivers were not present. A detailed auxiliary examination was positive for Epstein–Barr virus (EBV) DNA with 1.86 × 10^3^ copies/ml. EBV-specific antibody testing was positive for immunoglobulin M-viral capsid antigen (IgM-VCA), IgG-VCA, and the diffuse staining component of early antigen (EA-D). The levels of ALT (193 U/L) and AST (228 U/L) were elevated. The WBC, neutrophil, and PLT counts were normal, as well as the HB levels, and atypical lymphocytes were absent from the peripheral blood. Tests for common respiratory viruses were all negative. Moreover, tests for cytomegalovirus, *Toxoplasma gondii*, adenovirus, viral hepatitis, HIV, rubella virus, mycoplasma IgM, TPPA, fungal G test, GM test, bacterial cultures of blood and pharyngeal secretions, urine and stool examinations were all negative. We ruled out the possibility of tuberculosis or parasitic infections due to the lack of contact history, a BCG scar, and negative results for parasite-specific antibodies, PPD test, and T-SPOT. Tests for C-reaction protein (CRP), autoantibody, rheumatoid factor, anti-cyclic citrullinated peptide antibody, antineutrophil cytoplasmic antibodies (ANCAs, including p-ANCA and c-ANCA) and HLA-B27 were negative. Marrow cytology inspection did not suggest hemophagocytosis or other common blood system diseases. No other significant laboratory abnormalities were found after testing the renal, myocardial, and coagulation functions; in addition, blood gas analysis, and levels of blood glucose, blood ammonia, and blood lipids were normal. Echocardiography images showed aneurysms of the LCA (3.9 mm; *z* score, 5.01), LAD (2.4 mm; *z* score, 2.55), LCX (1.6 mm; *z* score, 0.69), and RCA (3.8 mm; *z* score, 6.37). We diagnosed the patient as having a primary EBV infectious mononucleosis based on her clinical features, the high loads of EBV-DNA, and the high levels of EBV-specific antibody, ALT, and AST. We also ruled out the possibility of KD after the lack of diagnostic criteria (9). Given that we also ruled out a coronary-pulmonary artery ﬁstula and the presence of systemic lupus erythematosus (SLE), we speculated that the EBV infection had caused the CALs (4, 10). The patient initiated a therapy with acyclovir and aspirin (3 mg/kg/day), bed rest, and symptomatic treatments. No glucocorticoids was not proposed since the clinical symptoms gradually resolved with negative EBV-DNA, normal liver function, and normal blood examination results one week later. The patient was discharged home with aspirin as her CALs remained to be unchanged by the repeated echocardiographic examination. Unfortunately, she did not have regular follow-ups in the outpatient clinic.

**Figure 1:**
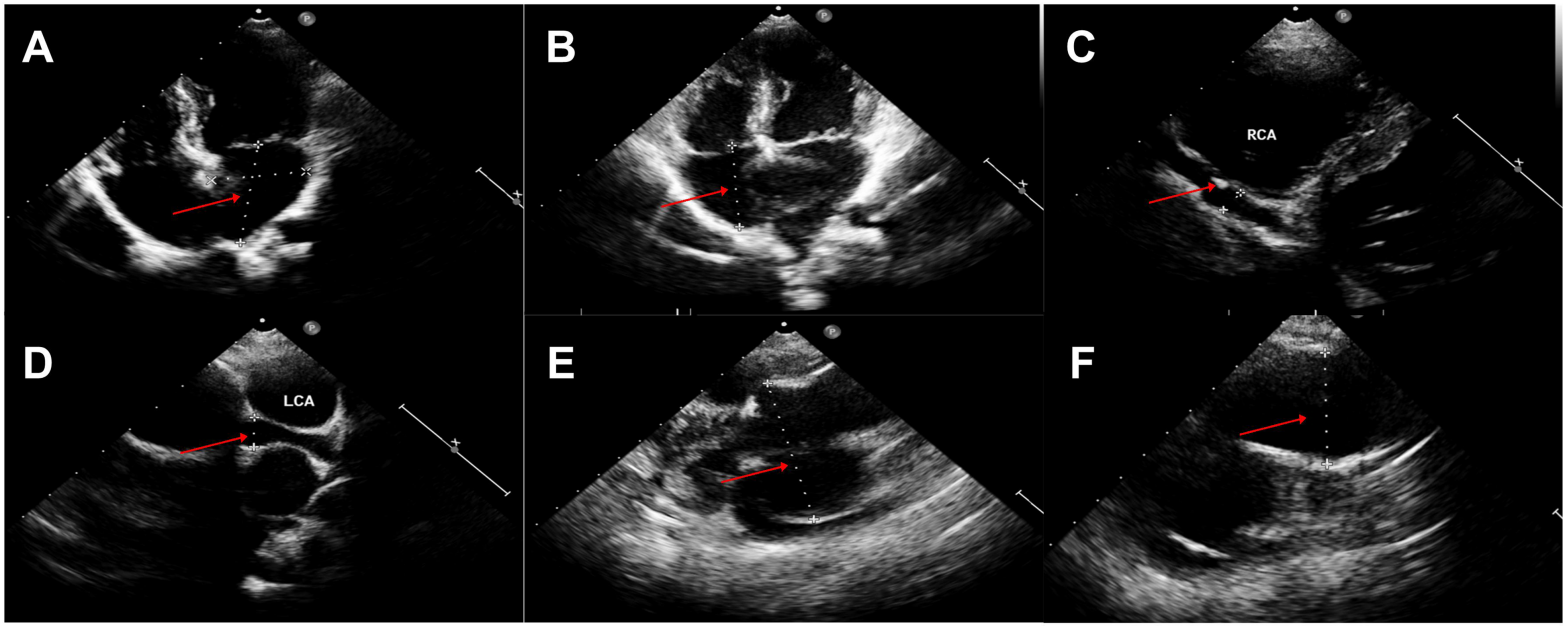
Echocardiography findings **(A,B)**. Enlarged atrium and ventricle (left atrium 44 × 35 mm, right atrium 38 mm, left ventricle 44 mm, right ventricle 16 mm), **(C)** Giant right coronary artery aneurysm (6.0 mm) and thrombosis (red arrow), **(D)** Left coronary artery aneurysm (9.5 mm), **(E)** Giant Valsalva sinus aneurysm (60 mm), **(F)** Ascending aorta dilation (35 mm).

**Figure 2:**
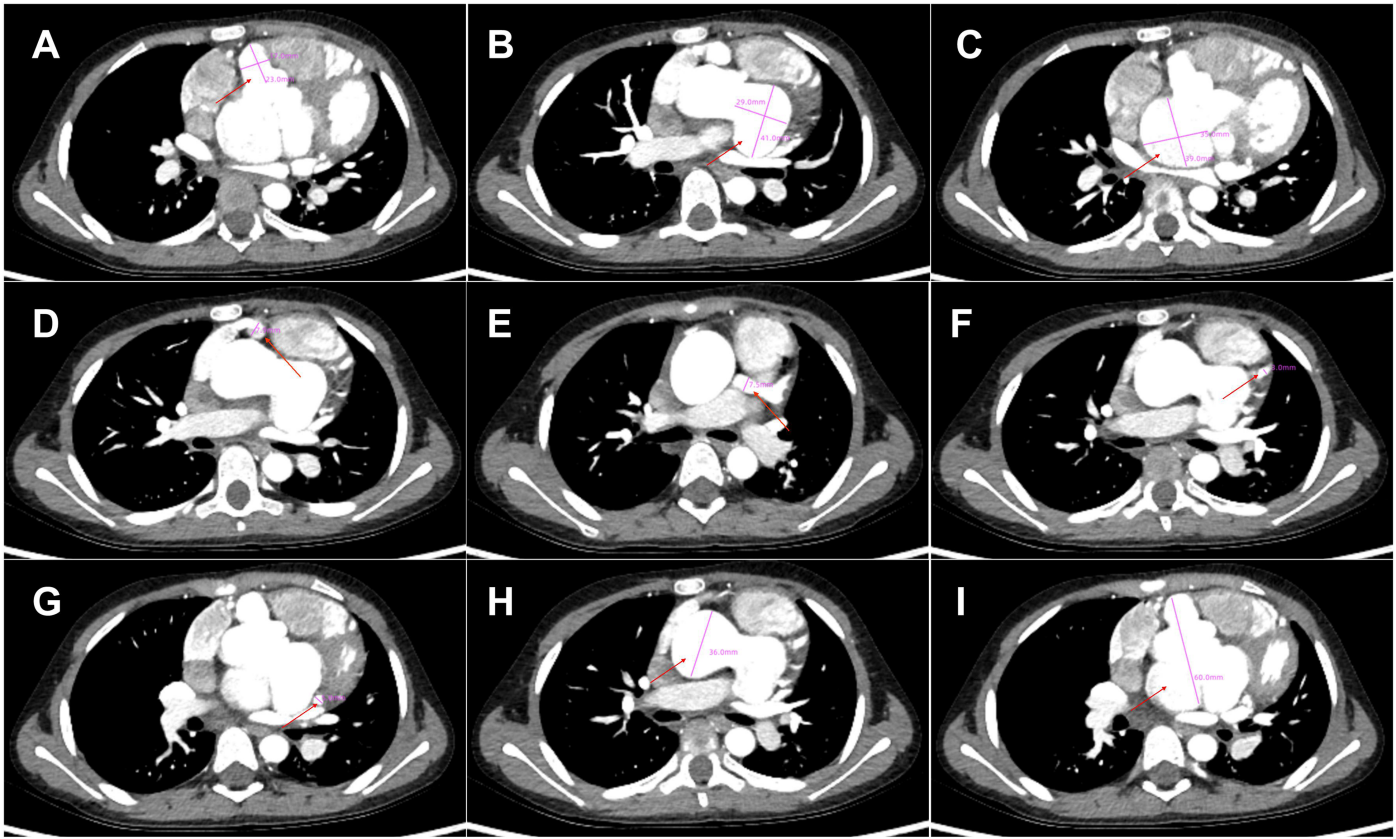
Cardiac computed tomography angiography findings. **(A)** Right coronary sinus aneurysm (23 mm × 17 mm, red arrow), **(B)** Left coronary sinus aneurysm (41 mm × 29 mm, red arrow), **(C)** Non-coronary sinus aneurysm (35 mm × 39 mm, red arrow) compressing the left atrium; **(D)** Right coronary artery aneurysm (7.9 mm, red arrow), thrombosis and calcifications in the distal right coronary artery; (**E–G**). Left main coronary artery aneurysm (LMCA, 7.5 mm), LAD (3 mm) and LCX (5 mm); **(H)** Dilated ascending aorta (36 mm, red arrow); **(I)**. Giant Valsalva sinus aneurysm (60 mm).

**Figure 3:**
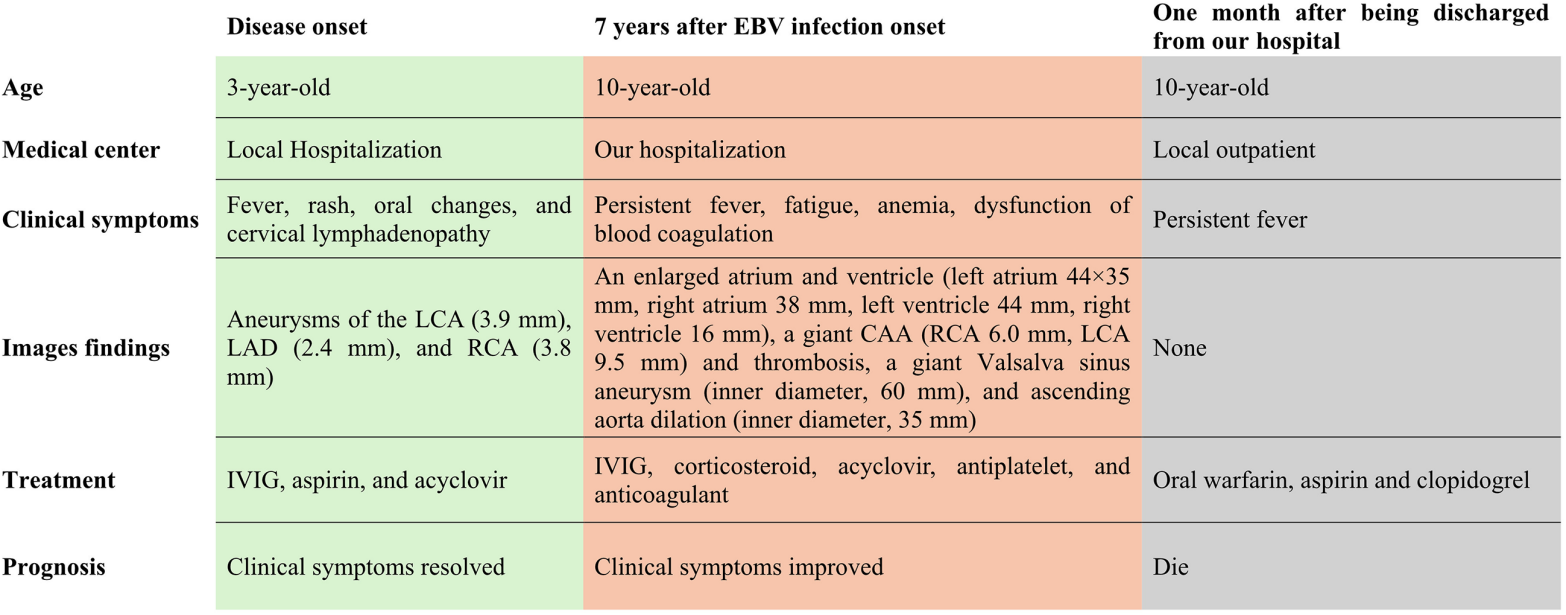
Clinical course of this patient.

Seven years later, the same girl, now 10 years old, was admitted to the local hospital with complaints of prolonged fever and fatigue. Her echocardiographic findings included giant left and right CAAs [LCA 9.0 mm (*z* score, 11.0), LAD 5.0 mm (*z* score, 5.91), LCX 5.0 mm (*z*-score, 6.1), RCA 16.3 mm (*z* score, 23.5)], a giant Valsalva sinus aneurysm, and an ascending aorta dilation. The patient was immediately admitted to our hospital again. Upon arrival, the physical examination revealed persistent fever, facial pallor, fatigue, ecchymosis can be seen in bilateral cubital fossa and abdominal wall, scattered petechiae can be seen on the skin of limbs, splenomegaly (line I, 10 cm; line Ⅱ, 10 cm; line Ⅲ, 0 cm), and hepatomegaly (6.5 cm below the costal margin), and no positive results were found in cardiac auscultation. The auxiliary examination showed 4.56 × 10^5^ copies/ml of Epstein–Barr virus (EBV) DNA. The patient had abnormal counts of WBCs (0.5 × 10^9^/L; normal range, 4.3–11.3 × 10^9^/L), neutrophils (0.35 × 10^9^/L; normal range, 1.6–7.8 × 10^9^/L), and lymphocytes (0.01 × 10^9^/L; normal range, 1.5–4.6 × 10^9^/L), and PLTs (10 × 10^9^/L; normal range, 100–450 × 10^9^/L) a low HGB level (73 g/L, normal range, 110–146 g/L), and high levels of CRP (53.78 mg/L), ALT (98 U/L), AST (159 U/L), and ferritin (11,276.20 ng/ml, normal range 10–291 ng/ml). Coagulation tests revealed 38.4 s for the activated partial thromboplastin time (APTT), 4.04 g/L of fibrinogen (Fg), and 1 mg/L of D-dimer (DDI). The levels of inflammatory cytokines including IL-6 (27.15 pg/ml; normal range < 20 pg/ml), IL-10 (79.12 pg/ml; normal range < 5.9 pg/ml), IL-2R (1,084 U/ml; normal range < 710 U/ml), IFN-a (28.65 pg/ml; normal range < 5.5 pg/ml), and sCD25 (19,534 pg/ml; normal range < 6,400 pg/ml) were elevated. The analysis of lymphocyte subgroups showed abnormally low numbers of T lymphocytes (CD3^+^, 0.01 × 10^9^/L), B lymphocytes (CD3^−^CD19^+^, <0.01 × 10^9^/L), and NK cells (CD3^−^CD56^+^CD16^+^, <0.01 × 10^9^/L). The triglyceride level (5.5 mmol/L; normal range < 1.7 mmol/L) was increased. The bone marrow biopsy revealed a hyperplasic bone marrow with hemophagocytosis. We ruled out other infectious diseases, autoimmune diseases, immunodeficiency, and tumors after obtaining negative results for tests on cytomegalovirus, *T. gondii*, adenovirus, viral hepatitis, HIV, mycoplasma IgM, TPPA, fungal G and GM, bacterial hemocultures, autoantibodies, humoral and cellular immunity, and tumor markers, as well as negative urine and stool examinations, and chest and abdomen CT images. The fundus and electrocardiographic examinations were also negative. This patient' medical history denied the EBV infection during her perinatal stage, organs transplantation, and blood products transfusion. This virus was also negative in her parents. In light of our findings (EBV infection, fever, splenomegaly, bicytopenia, hypertriglyceridemia, hypofibrinogenemia, hemophagocytosis, ferritin ≥ 500 µg/L, low NK-cell activity, and soluble CD25 ≥ 2,400 U/m), we consulted with an expert in infectious diseases and an expert in blood diseases to diagnose a chronic active EB virus infection (CAEBV) complicated with hemophagocytic lymphohistiocytosis (HLH), on the basis of the diagnostic criteria on the guideline for diagnosis and treatment of CAEBV in children and the HLH 2004 clinical trial (11–13). We ruled out the possibility of familial HLH and inherited connective tissue disease with a negative whole exome sequencing and the existence of an unaffected sibling. Unfortunately, the patient's guardian abandoned the standard therapy of EBV-HLH due to economic difficulties and agreed only to a treatment with IVIG, corticosteroid, acyclovir, antiplatelet, and anticoagulant. A high dose of IVIG (2 g/kg as a single dose) and the methylprednisolone (20 mg/kg/day) prescription were initiated, with gradual tapering of the methylprednisolone to oral prednisone (2 mg/kg/day) after the patient's fever resolved and the levels of WBC, HB, PLT, and inflammatory cytokines became normal. An echocardiographic examination revealed an enlarged atrium and ventricle (left atrium 44 × 35 mm, right atrium 38 mm, left ventricle 44 mm, right ventricle 16 mm), a giant CAA (RCA 6.0 mm [*z* score, 8.55]; LCA 9.5 mm [*z* score, 11.64]) and thrombosis, a giant Valsalva sinus aneurysm (inner diameter, 60 mm), and ascending aorta dilation (inner diameter, 35 mm) (Figure 1). A cardiac computed tomography angiography (CCTA) further showed an enlarged left ventricle, a dilated ascending aorta (36 mm), a right coronary sinus aneurysm (23 mm × 17 mm), a non-coronary sinus aneurysm (35 mm × 39 mm) compressing the left atrium, a left coronary sinus aneurysm (41 mm × 29 mm); an RCA aneurysm [7.9 mm (*z* score, 11.86)], thrombosis and calcifications in the distal right coronary artery; a left main coronary artery aneurysm [LMCA, 7.5 mm (*z* score, 9.19)], a LAD [3 mm (*z* score, 2.51)], and an LCX [5 mm (*z* score, 6.13)] (Figure 2); and absence of coronary-pulmonary artery fistulas. We attributed these cardiac complications to the vasculitis produced by the CAEBV after the detailed discussion among the members of the Multiple Disciplinary Team (MDT) comprised of experts in infectious, blood, autoimmune, and cardiac diseases of our hospital. Accordingly, we prescribed a subcutaneous injection of low molecular weight heparin calcium (LMWHC) and sequential therapy with oral warfarin, combined with aspirin and clopidogrel. Three weeks later, the patient was finally discharged home. Unfortunately, one month later, the fever attacked this patient. Owning to economic difficulties, the patient's guardian had refused further treatment and took the patient home. During our subsequent follow-up visit, the girl subsequently passed away (Figure 3).

## Discussion

Although primary EBV infection in children is usually asymptomatic and self-limiting with a favorable prognosis (2), some patients with an immune deﬁciency or impairment develop CAEBV, HLH, and tumors. EBV-HLH is a high-mortality, high-morbidity disease with life-threatening complications. Approximately 8.5% of patients with CAEBV present with CALs (4); whereas the incidence of CALs in KD is approximately 4% after IVIG treatment (5). In addition to CALs, severe EBV infections may cause myocarditis, valvular heart disease, heart failure, and PAH (6). However, we found only two reports of patients with CAEBV presenting with Valsalva sinus involvement at the onset of their disease (7, 8). To the best of our knowledge, this is the first report of a pediatric case of EBV infection that progressed to CAEBV and EBV-HLH and developed fatal cardiovascular complications seven years after the disease onset, presenting with giant CAA and thrombosis, giant Valsalva sinus aneurysm, and ascending aorta dilation. Clinicians need to be aware of the potential cardiac complications in patients with EBV infection, especially in those with CAEBV and/or EBV-HLH.

On the pathogenesis mechanism of cardiovascular complications involving EBV infection, there is ample evidence showing how EBV directly damages the heart, and viral genome analyses by quantitative PCR have shown EBV-DNA copies in endomyocardial biopsies (EMB) from patients with cardiomyopathy (14, 15). The EMB from a patient with ongoing perimyocarditis was shown to contain high numbers of EBV-encoded RNA copies in CD8^+^ T-lymphocytes, demonstrating that the EBV infection contributed to a severe, chronic active infection in the myocardium (16). Animal myocarditis models have shown that EBV-associated cardiomyocytes negative for EBV-DNA copies still presented a severe inflammatory infiltration (17). Moreover, myocardial necrosis was not observed in B- and T-lymphocyte-deﬁcient mice with high EBV-DNA copy numbers, suggesting that the myocardial injury might not have completely been attributed to viral replication within the cardiac tissues (17). EBV-related coronary artery lesions associated with lymphoid vasculitis are considered a kind of immune injury (18) due to chemotaxis, recruitment, adhesion, infiltration, cytotoxic injury, and cytokine secretion by the local inflammatory cells (15). EBV can induce local inflammatory infiltration by infecting T cells, natural killer (NK) cells, or B cells (19). Cytotoxic T cells (CTLs) infected with EBV produce excessive amounts of vascular endothelial growth factor (VEGF), which enhances the post-capillary permeability of veins and venules, further promoting vascular wall degradation (20). In addition, vascular lesions may be attributed to adhesion molecules and cytokines secreted by EBV-positive NK/T cells (20). EBV also causes vascular endothelial damage via the production of deoxyuridine triphosphatase (dUTPase) during the viral replication, subsequently increasing interleukin-6 (IL-6) levels (21, 22). Therefore, it was postulated that the infiltration of EBV-infected T- and NK-cells and the related inflammatory reactions mainly accounted for cardiac complications during the progressive disease course. Cardiac complications in patients with CAEBV tend to be associated with high EBV load in peripheral blood samples, rather than with the type of CAEBV or the time interval from disease onset to the development of cardiac disease (23). Muneuchi et al. have found the CAEBV patients with cardiac complications presented fever more frequently than the patients without complications. It lends itself to reason, therefore, a high EBV load, fever, and cytopenias might be risk factors for the development of coronary artery lesion (23). Furthermore, more studies should be carried out to explore the risk factors of cardiac complications in patients with EBV infections. In the case of our patient, she developed CAEBV and EBV-HLH indicating a severe hyper-inflammatory reaction, which was probably responsible for her serious cardiovascular complications. As mentioned, we speculate that our patient's cardiovascular complications might be attributed to direct virus infiltration and immune injury. However, the pathogenesis of cardiovascular complications involving EBV infection remain incompletely understood and advanced animal and cell model studies still need to clarify the relevant mechanisms.

Coronary artery lesions are the most common cardiovascular complications in patients with CAEBV (4). In a nationwide survey, the incidences of coronary artery aneurysms and myocarditis in patients with CAEBV were respectively 8.5% and 6% (4). In children with CAEBV, cardiovascular complications are a significant mortality risk factor (23). As shown in the Table 1, patients with EBV infection shared the similar clinical characteristics, namely younger age of disease onset, prolonged fever (7, 8, 18, 24–26), rash (7, 18, 27), and high EBV–DNA load (7, 8, 26). In addition, most patients with severe cardiac complications were found on follow–up, which indicated that the younger age of disease onset, prolonged fever, and high EBV–DNA load were risk factors for cardiac complications in those patients (7, 8, 24, 26–28). Reports of aortic and aortic branch involvements have been uncommon: abdominal aorta (18, 24, 25, 27, 28), carotid artery (25), subclavian artery (25), common iliac artery (24–26), and thoracic aorta (8, 18). Notably, ours seems to be the third case of CAEBV involving the Valsalva sinus (7, 8). Y. Sato et al. reported the case of an 11-year-old girl diagnosed as having CAEBV complicated with Valsalva sinus dilatation, her pathological findings showed fibroid necrotic changes with inﬁltration of a few abnormal lymphoid cells in the wall of the Valsalva sinus (7). Qirui Li et al. presented the case of a 5-year-old girl with CAEBV complicated with multiple arterial aneurysms mainly involving the right coronary artery sinus, left main coronary artery, aorta, and its major branches (8). Notably, the cardiovascular complications developed during the initial stages of the disease in the reports by Y. Sato and Li; whereas our patient presented a seven-year gap between the initial. Infection and the development of the fatal cardiovascular complications. Moreover, her giant Valsalva sinus aneurysm involved simultaneously the right and left coronary sinuses, and the non-coronary aneurysm. In addition, her giant non-coronary sinus aneurysm compressed the left atrium. At the same time, our patient had a giant CAA and thrombosis, and an ascending aorta dilation. Congenital Valsalva sinus aneurysms are caused by connective tissue diseases, such as those in Apert's, Marfan's, and Ehlers-Danlos' syndromes (29–31). By contrast, acquired Valsalva sinus aneurysms can be found in connective tissue disease, infectious etiologies (syphilis, bacterial endocarditis, and tuberculosis), vasculitis diseases (Takayasu's Arteritis), chronic atherosclerotic changes, medial cystic necrosis, chest trauma, and iatrogenic injury during aortic valve surgery (32–37). Thus, clinicians should know that severe EBV infection, especially for CAEBV and/or EBV-HLH, may also cause Valsalva sinus lesions besides the coronary artery and aorta lesions. Moreover, clinicians need to be aware of these complications during the long-term follow-up of patients with EBV infection, especially for patients with CAEBV and/or EBV-HLH.

**Table 1:** Summary of patients with CAEBV involving coronary artery, valsalva sinus, and aorta.

Author, year	Sex	Onset age (years)	Diagnosis	Cardiovascular complications	Treatment	Prognosis
Ours	F	3	CAEBV EBV-HLH	CAA, giant valsalva sinus aneurysm, ascending aorta dilation	IVIG, warfarin, aspirin, and clopidogrel	Dead
Li Q, 2022 (8)	F	5	CAEBV	CAA; giant valsalva sinus aneurysm; heart valve disease	Aspirin, dipyridamole, warfarin and metoprolol	Dead
Ba H, 2019 (27)	M	9	CAEBV	CAA; the aortic sinus dilation; the bilateral pulmonary dilation; minor abdominal aortic stem and the distal section of the superior mesenteric artery dilatation	Prednisolone, cyclophosphamide, sildenafil and bosentan	Pulmonary hypertension improved
Nakagawa A, 1996 (8)	F	5	CAEBV	CAA; thoracic and abdominal aortic aneurysms; Valsalva Sinus aneurysm	Acyclovir and interferon	Dead
Xiao H, 2020 (28)	F	5	CAEBV	CAA; thickened aortic wall; segmental abdominal aorta dilation and stenosis	HSCT	Her CAAs did not progress,and uveitis was well controlled
Kang R, 2020 (24)	M	42	CAEBV	CAA; abdominal aortic aneurysm; bilateral common iliac artery aneurysms	HSCT	Further dilation of aneurysms
Murakami K, 1998 (25)	F	10	CAEBV	CAA; bilateral common carotid and subclavian arteries, abdominal aorta and its major branches, and bilateral common iliac arteries were involved, and all showed aneurysmal dilation of the lumens.	Steroids and immunosuppressive drugs	Dead
Jiang S, 2016 (26)	F	16	CAEBV	Aneurysms involving the aorta and its major branches; multiple aneurysms and stenoses of the coronary arteries	Medrol and Leflunomide	New-onset mural thrombi of the bilateral common iliac artery; new-onset occlusion of the right coronary artery
Sato Y, 2006 (7)	F	11	CAEBV	Valsalva sinus dilation and aortic regurgitation.	HLH-94	Dead

Abbreviations: CAEBV, chronic active Epstein–Barr virus infection; HLH, hemophagocytic lymphohistiocytosis; CAA, coronary artery aneurysm; HSCT, hematopoietic stem cell transplantation; IVIG, intravenous immunoglobulin.

Regarding the management of cardiovascular complications associated with EBV infection, medical therapy should be provided according to the types of cardiovascular complications (38, 39). Moreover, surgical repair should be considered for patients with ruptured or symptomatic non-ruptured Valsalva sinus aneurysm since this condition can be fatal (40, 41). In any case, cardiovascular complications are mostly observed in patients with CAEBV, and the cornerstone of therapy should be to address the CAEBV. Unfortunately, the effectiveness of antiviral therapy is limited. Allogeneic hematopoietic stem cell transplantation (HSCT) is often curative for CAEBV disease, and the treatment should be considered early in the course of the disease as patients are more likely to tolerate the procedure then (42). The conventional treatment (including rituximab, immunosuppressive therapy, cytotoxic chemotherapy, and autologous CTLs) may yield a temporary remission, but the effect of these agents is not curative (43). Chemotherapy is used to reduce viral load and control disease activity in CAEBV before performing HSCT. In addition, chemotherapy could reduce the risk of complications related to HSCT, and may control the disease activity of CAEBV, which contributes to improving the outcome of HSCT (3). In this case, due to financial constraints, the patient's guardian declined the optimal treatment plan. As an alternative, we administered high-dose IVIG and glucocorticoids to suppress the inflammatory cytokine storm, while also providing antiviral and supportive care, in order to save the patient's life to the greatest extent possible.

## Conclusion

We reported the rare case of a pediatric patient with EBV infection progressing to CAEBV/EBV-HLH. The patient developed uncommon and fatal cardiovascular complications seven years after the onset of the initial infection. Clinicians need to be aware of the possibility of these long-term EBV infection complications during the follow-up of patients, especially in those with CAEBV and/or EBV-HLH.

## Data Availability

The original contributions presented in the study are included in the article/Supplementary Material, further inquiries can be directed to the corresponding authors.
